# Supplementary material for: Individualized epidemic spreading models predict epilepsy surgery outcomes: A pseudo-prospective study
Source: Netw Neurosci. 2024 Jul 1;8(2):437–65. doi: 10.1162/netn_a_00361 (PMC11142635; doi:10.1162/netn_a_00361)
Supplement: Supplementary file 1 [file netn-8-2-437-s001.pdf]

Supplementary information to: Individualized epidemic spreading models predict epilepsy surgery outcomes: a pseudo-prospective study

Ana. P. Millán[1,2,*], Elisabeth C.W. van Straaten[1,5,6], Cornelis J. Stam[1,4,6], Ida A. Nissen[1], Sander Idema[3,5,7], Piet Van Mieghem[8], and Arjan Hillebrand[1,4,5]

[1]Amsterdam UMC location Vrije Universiteit Amsterdam, Department of Clinical Neurophysiology and MEG Center, De Boelelaan 1117, Amsterdam, The Netherlands
[2]Institute "Carlos I" for Theoretical and Computational Physics, and Electromagnetism and Matter Physics Department, University of Granada, E-18071 Granada, Spain
[3]Amsterdam UMC, Vrije Universiteit Amsterdam, Department of Neurosurgery, De Boelelaan 1117, Amsterdam, The Netherlands
[4]Amsterdam Neuroscience, Brain Imaging, Amsterdam, The Netherlands
[5]Amsterdam Neuroscience, Systems & Network Neurosciences, Amsterdam, The Netherlands
[6]Amsterdam Neuroscience, Neurodegeneration, Amsterdam, The Netherlands
[7]Amsterdam Neuroscience, Cancer Biology and Immonology, Amsterdam, The Netherlands
[8]Faculty of Electrical Engineering, Mathematics and Computer Science, Delft University of Technology, Delft, The Netherlands
[*]Corresponding author: a.p.millanvidal@amsterdamumc.nl

1 Patient data

Basic statistics of the patient groups are summarized in Supp. tables 1 (modeling cohort) and 2 (validation cohort).

2 Individualized MEG brain networks

Seizure propagation was modeled on the patient-specific brain networks, as derived from MEG data, for both cohorts. One to three eyes-closed resting-state (supine position) MEG recordings of 10 to 15 minutes each were acquired for each patient in a magnetically shielded room (Vacuumschmelze GmbH, Hanau, Germany) during routine presurgical clinical practice (whole-head MEG system (Elekta Neuromag Oy, Helsinki, Finland) with 306 channels consisting of 102 magnetometers and 204 gradiometers). The data were sampled at 1250 Hz, and online filtered with an anti-aliasing filter at 410 Hz and a high-pass filter of 0.1 Hz. The head's position relative to the MEG sensors was determined using the signals from 4 to 5 head-localization coils that were recorded continuously. The positions of the head-localization coils and the outline of the scalp (roughly 500 points) were measured with a 3D digitizer (Fastrak, Polhemus, Colchester, VT, USA). The temporal extension of Signal Space Separation (tSSS) [1, 2] was used to remove artifacts using Maxfilter software (Elekta Neuromag, Oy; version 2.1), and the MEG data were filtered in the broadband ($0.5 - 48.0$ Hz). For a detailed description and parameter settings see Hillebrand et al. [3].

Pre-operative MRI scans (8 channel phased-array head coil on a 3T whole-body MR scanner, Discovery MR750, GE Healthcare, Milwaukee, Wisconsin, USA) were used for co-registration with the MEG data. Anatomical 3D T1-weighted images were obtained with a fast spoiled gradient-recalled echo sequence, and were interpolated to 1 mm isotropic resolution during reconstruction. The points on the scalp surface were used for co-registration of the MEG scans with the anatomical MRI of the patient through surface-matching. A single sphere was fitted to the outline of the scalp and used as a volume conductor model for the beamforming approach.

Neuronal activity was reconstructed using an atlas-based beamforming approach, modified from Hillebrand et al. [4], to reconstruct the time-series of neuronal activation of the ROI centroids [5]. We considered the 246 ROIs of the Brainetome (BNA) atlas [6], the centroids of which were inversely transformed to the co-registered MRI of the

Case	Sex	Resection Area	S_{RA}	Engel Score	#E	#ECP	N_{SR}
P1	F	RF	4	1A	13	128	47
P2	F	RT, RO	13	1A	14	142	50
P3	F	LT, LO	5	1A	15	144	53
P4	M	RT	13	1A	13	126	49
P5	F	RT	10	1A	11	109	42
**P6**	**F**	**RT**	**5**	**2A**	**9**	**99**	**40**
P7	F	LT	5	1A	11	110	44
P8	F	LP	4	1A	10	104	37
P9	M	RT, RI, RP	3	1A	12	102	38
**P10**	**F**	**RT**	**13**	**2D**	**11**	**114**	**45**
**P11**	**F**	**LF**	**4**	**2C**	**13**	**117**	**47**
P12	M	LF	6	1A	12	124	40
P13	M	LT	5	1A	12	106	30
**P14**	**F**	**LT**	**6**	**3A**	**15**	**194**	**60**
P15	M	RT	12	1A	10	107	32

Supp. Table 1: Patient data (modellng cohort). Ep. = Epilepsy, y = years, S_{RA} = number of resected ROIs, #E = number of intracranial electrodes, #ECP = total number of electrode contact points, N_{SR} = number of brain regions sampled by the SEEG electrodes. F = female (under "Sex"), M = male, R = right, L = left, F = Frontal lobe (under "Resection Area"), T = Temporal lobe, O = Occipital lobe, I = Insula, P = Parietal lobe.

patient. Then, a scalar beamformer (Elekta Neuromag Oy; beamformer; version 2.2.10) was applied to reconstruct each centroid's time-series, as detailed elsewhere [5]. The time-series of each centroid were visually inspected for epileptiform activity and artifacts. On average, 59.08 (range: $48 - 83$) 16384-sample interictal epochs of sufficient quality were selected for each patient. The epochs were further analyzed in Brainwave (version 0.9.151.5 [7]) and were down-sampled to 312 Hz, and filtered in the broadband (0.5 - 48 Hz).

3 iEEG Propagation Pattern

Patients in the modeling cohort underwent invasive EEG recordings using stereotactic electrode implantation as described also in [8]. The number and location of the intracerebral electrodes (Ad-Tech, Medical Instrument Corporation, USA, 10-15 contacts, 1.12 mm electrode diameter, 5 mm intercontact spacing; and DIXIE, 10-19 contacts, 0.8 mm electrode diameter, 2 mm contact length, 1.5 insulator length, $16 - 80.5$ insulator spacer length) were planned individually for each patient by the clinical team, and were based on the location of the hypothesized SOZ and seizure propagation pattern. The number of electrodes per patient varied between 9 and 15 (average $= 12.1 \pm 1.8$) and the total number of contacts between 194 and 99 (average $= 121 \pm 24$).

The locations of the electrode contact points were obtained for each patient from the post-implantation CT scan (containing the iEEG electrodes) that was co-registered to the preoperative MRI scan using FSL FLIRT (version 4.1.6) 12 parameter afine transformation. Each contact point was assigned the location of the nearest BNA ROI centroid. Given that BNA ROIs are in general larger than the separation between contact points, different contact points could be assigned to the same BNA ROI.

An iEEG seizure pattern was derived for each patient by a clinician (ECWvS). First, a representative seizure was chosen for each patient. Then, the onset time of ictal activity was identified for each iEEG channel, and the channels were sorted according to their onset times. In case two channels became active at the same time, they were assigned the same activation order. This activation pattern was then translated into the BNA space, so that the each sampled ROI i was assigned an activation step. If channels with different onset times belonged to the same BNA ROI, the ROI was assigned the earliest of the possible times. This constituted the *iEEG seizure pattern.*

4 Seed-probability maps

In order to characterize the seed-probability maps, in Supp. figure 1 we show the fraction of seed-probability accounted for by RA nodes (panels A and E), the fraction of nodes with non-zero seed-probability that belonged to the RA

Case	Sex	Resection Area	S_{RA}	Engel Score
1	F	RT	5	1A
**2**	**M**	**LT**	**15**	**2A**
3	M	RT	10	1A
4	F	LT	22	1A
5	M	LT	3	1A
**6**	M	**RT**	**11**	**2B**
7	M	RF	2	1A
8	F	RT	12	1A
**9**	**M**	**LF LP**	**3**	**4B**
10	F	RF	4	1A
11	M	RT	12	1A
12	M	RT	11	1A
**13**	**M**	**LP**	**5**	**1B**
14	F	LT	18	1A
15	M	RT	10	1A
16	M	RT	18	1A
17	F	LT	19	1A
18	M	RT	3	1A
19	M	LT	4	1A
20	M	LT	12	1A
**21**	**M**	**RF**	**10**	**3A**
22	F	LT	19	1A
23	F	RT	12	1A
24	F	LT	17	1A
25	M	LT	15	1A
**26**	**M**	**LT**	**13**	**2B**
27	M	LT	10	1A
28	F	LT	3	1A
29	F	LT	10	1A
**30**	**M**	**RT**	**17**	**4A**
31	M	RT	19	1A
32	F	LP	2	1A
**33**	**F**	**LT**	**2**	**1B**
34	M	LT LP	5	1A

Supp. Table 2: Patient data (validation cohort). S_{RA} = number of resected ROIs, F = female (under "Sex"), M = male, R = right, L = left, F = Frontal lobe (under "Resection Area"), T = Temporal lobe, O = Occipital lobe, I = Insula, P = Parietal lobe.

(panels B and F) and the comparison of the RA seed-probability (panels C and G) and node (panels D and G) fractions between the SF and NSF groups, respectively for the modeling and validation cohort.

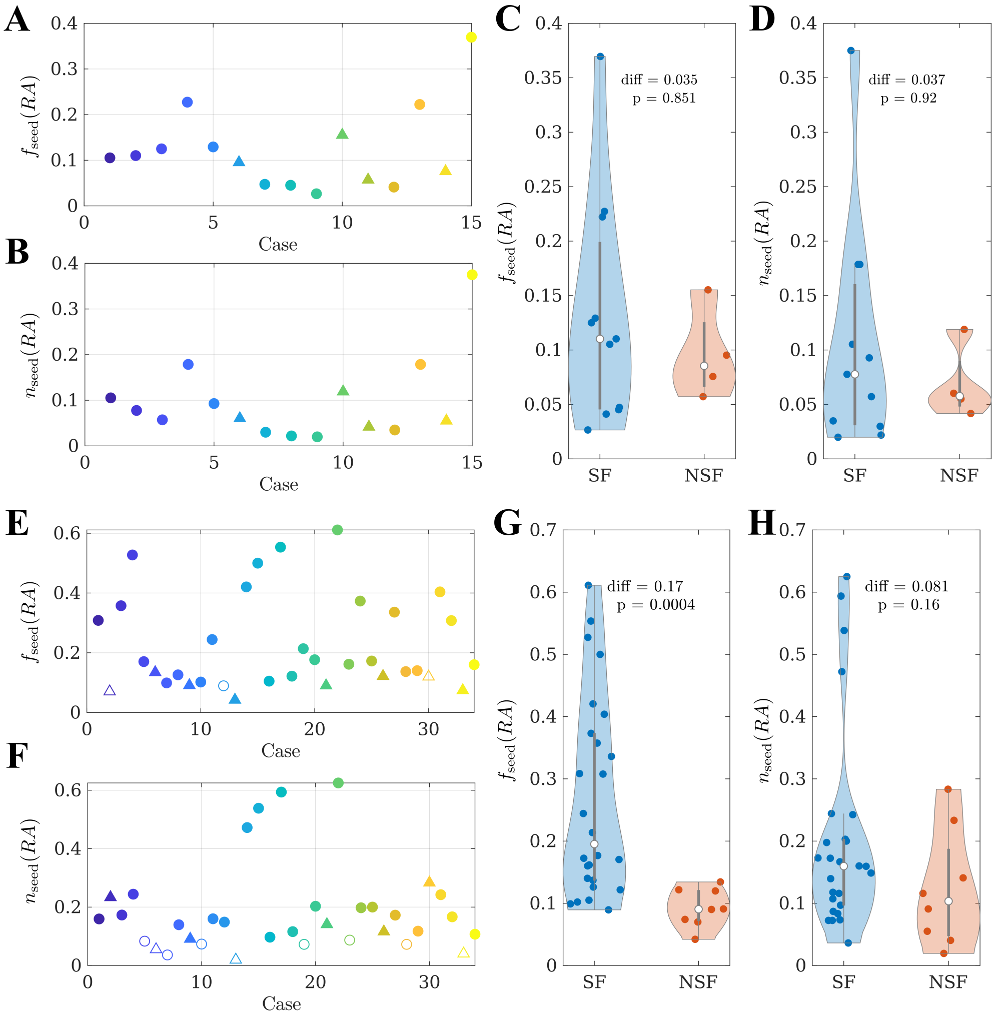

Supp. Figure 1: Characterization of seed-probability maps, modeling (top) and validation (bottom) cohorts. **A** and **E** Fraction of the total seed-probability in RA nodes, $f_{\mathrm{seed}}(RA)$, for each patient. Filled markers indicate that the seed-probability in the RA nodes was larger than expected by chance (this occurred for all patients in the modeling cohort). **B** and **F** Fraction of nodes with non-zero seed-probability that belonged to the RA, $n_{\mathrm{seed}}(RA)$. In **A, B, E** and **F**, SF (NSF) patients are indicated by circular (triangular) markers. The colorcode indicates the patient, also given by the x-axis. Filled markers indicate that there were more RA nodes with non-zero seed-probability than expected by chance. **C,D** and **G,H** Comparison of the RA seed-probability (**C,G**) and node (**D,H**) fractions between the SF and NSF groups using a two-sided Wilcoxon ranksum test (panel C: diff=0.035, $p = 0.85$, ranksum $rks = 90$; panel D: diff=0.037, $p = 0.92$, $rks = 89$, panel G: diff=0.17, $p = 4 \cdot 10^{-5}$, $rks = 542$; panel H: diff=0.081, $p = 0.16$, $rks = 489.5$).

5 Seizure propagation model

5.1 SIR Dynamics

The discrete-time SIR model [9, 10] was used to simulate the propagation of ictal activity from a set of *seed* nodes (or brain regions) that were set to be infected (or in the ictal state) at the beginning of the simulation. The SIR dynamics considered here are characterized by two parameters: the probability β_{ij} that each infected node i propagates the infection to a neighbour j ($S \to I$) and the probability γ_i that each infected node i recovers ($I \to R$). For simplicity, and due to the limited amount of data available (one iEEG-recorded seizure per patient, and $N = 15$ patients) we considered here a global recovery rate $\gamma_i = \gamma$ and spreading probabilities given by the MEG network structure: $\beta_{ij} = w_{ij}$. As discussed in our previous studies [8, 11], the iEEG seizure patterns only accounted for the propagation of ictal activity and not the recovery to the healthy state, thus considering local recovery rates is beyond the scope of this study. In this manner we avoided introducing arbitrary time scales (i.e. to account for the duration of the ictal state) into the seizure propagation model.

The SIR dynamics was simulated by an adaptive Monte Carlo method (the BKL algoritihm) in Matlab in discrete time, such that at each time step one (and only one) new node became infected, and they were iterated $N_R = 10^4$ times per set of (ρ, γ) parameters.

5.2 Parameter fit: the ESSES model

The two control parameters of ESSES are the density of connections in the network, ρ, indirectly setting the spreading probabilities, and the global recovery rate γ. ρ and γ were fitted in the retrospective study (modeling cohort) by comparing the spatio-temporal propagation pattern of the ESSES-modeled seizures to the patient's iEEG seizure pattern (constructed as described above), when the resection area was used as the seed for epidemic spreading. For each set of ESSES model parameters, we measured the spatio-temporal seizure propagation profile described by the probability $p_i(t)$ that each ROI i becomes infected at step t. Then, mean infection time t_i of each ROI i is defined as

$$t_i = \sum_{t=0}^{T} p_i(t), \tag{1}$$

where T is the maximum integration time. t_i was then sub-sampled to the ROIs sampled by the iEEG electrodes, and each of these ROIs was assigned an *infection order* according to t_i. This constituted the *ESSES seizure pattern*.

The *goodness-of-fit* $C(\rho, \gamma)$, quantified how similar the ESSES and iEEG seizure-propagation patterns were, and it was measured following the same procedure as in Ref. [8]. It took into account two factors: the weighted correlation between infection orders of ROIs that were infected in both patterns, C_w, and the overlap between the infected (or active) and susceptible (or inactive) ROI sets of both patterns, P_{overlap}, i.e.

$$C = C_w \cdot P_{\text{overlap}}. \tag{2}$$

In order to take into account the stochastic nature of the SIR dynamics, C_w was weighted by the fraction of realizations that each ROI i got infected during a modeled seizure, $P_{IR}(i)$. Similarly, P_{overlap} was weighted by $P_{IR}(i)$ as:

$$P_{\text{overlap}} = N_{\text{iEEG}}^{-1} \left[\sum_{i \in \mathbb{S}} P_{\text{IR}}(i) + \sum_{i \in \mathbb{H}} (1 - P_{\text{IR}}(i)) \right] = P_{\text{act}} + P_{\text{inact}}, \tag{3}$$

where N_{iEEG} is the number of ROIs sampled by the iEEG electrodes (on average $= 43.6 \pm 7.9$), and $\mathbb{S}$ and $\mathbb{H}$ are respectively the sets of active (in the seizure state) and inactive (in the healthy state) ROIs in the iEEG pattern. The total correlation C equals 1 in case of exactly equal activation patterns, 0 in the case of null-overlap or correlation, and -1 in the case of complete anti-correlation of activation times (but equal seizure areas). We note however that C decays from 1 faster than a simple correlation metric, since it takes into consideration not only the activation times, but also the activation areas.

In order to fit ρ and γ, we measured C for a range of values ρ and γ logarithmically distributed (between 0.01 and 0.35 for ρ and between 0.01 and 1.00 for γ), considering the resection area as the seed of seizure propagation [8, 11]. For each parameter configuration we performed 10 iterations (each over $N_R = 10^4$ iterations of the SIR dynamics), to obtain average C values and their fluctuation for each patient. We then found the parameter set that maximized C for each patient (individual model fit) and at the group level (population model fit). The population model fit was

used for all subsequent analyses in the main text (for both the modeling and validation cohorts). As in our previous study [8], we found that the population model fit led to a better classification between the SF and NSF groups, likely due to a reduced influence of noise.

The results for the individual model fit are summarized in Supp. figure 2 (on average, $C = 0.12$ with standard deviation $\text{std}_C = 0.07$). The model provided a (not significantly) better fit for the SF ($C_{SF} = 0.14 \pm 0.08$) than NSF ($C_{\text{NSF}} = 0.08 \pm 0.02$) patient groups ($C_{SF} - C_{\text{NSF}} = 0.05$, ranksum rks= 99, $p = 0.18$, exact two-sided Wilcoxon ranksum test), as shown in Supp. figure 2A. A ROC classification analysis based on the goodness-of-fit returned a good classification result with an area under the curve (AUC) of $AUC = 0.750$ (Supp. figure 2B). The confusion matrix corresponding to the optimal point of the ROC curve, according to the Youden criterion (to control for the class imbalance), is shown in 2C. We found an accuracy of 0.80, precision = 0.60, sensitivity = 0.75 and $F1 = 0.67$. There were no significant differences in the fit parameters ρ and γ between the groups.

Supp. figure 3 shows the results of the model fit at the group level for the modeling cohort. The details of the statistical comparison of the model fit results between the seizure-free and non-seizure-free groups are shown in Supp. table 3.

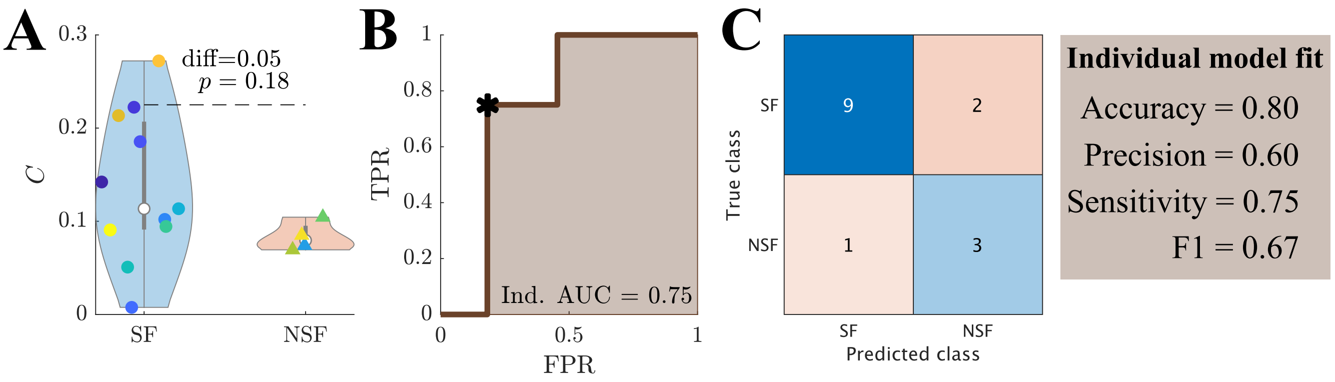

Supp. Figure 2: Seizure propagation model: model fit results. Group comparison of the goodness-of-fit using the individual model fit. **A** Violin plots of the goodness-of-fit distributions for each group. The significance analysis was performed with an exact two-sided Wilcoxon rank test. **B** ROC (receiving-operator-characteristic) curve analyses, where TPR and FPR respectively indicate the true positive (NSF patient classified as NSF) and false positive (SF patient classified as NSF) rates. **C** Confusion matrix corresponding to the optimal operating point of the ROC curve in panel B (shown by a black asterisk).

	Cohort	SF	NSF	diff	rks	p
$\bar{C}$	Modeling	0.08 ± 0.08	-0.02 ± 0.08	0.11	72	0.04

Supp. Table 3: Summary of statistical comparisons: difference in goodness-of-fit between SF and NSF groups (modeling cohort).

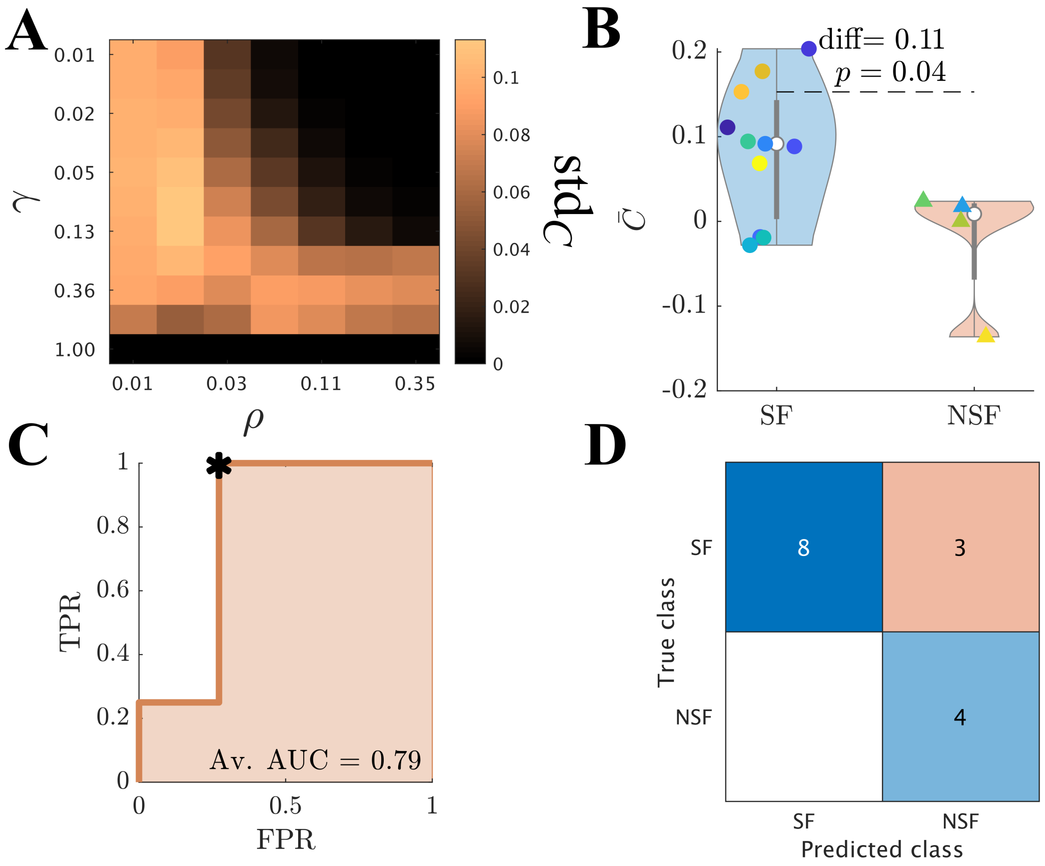

Supp. Figure 3: ESSES parameter fitting (modeling cohort). **A** $C_{std}(\rho, \gamma)$ map displaying the standard deviation of the model fit C for the modeling cohort. **B** Violin plots of the goodness-of-fit distributions for the SF and NSF groups. **C** ROC (receiving-operator-characteristic) curve analysis, where TPR and FPR respectively indicate the true positive (NSF patient classified as NSF) and false positive (SF patient classified as NSF) rates. The optimal classification point (Youden criterion) is shown by a black asterisk, and the corresponding confusion matrix is shown in panel **D**. The confusion matrix indicates the number of SF and NSF cases that were correctly (diagonal elements) and incorrectly (off-diagonal elements) classified.

5.3 Alternative resection strategies

We used an optimization method based on simulated annealing to find alternative resection strategies R of increasing size $S(R)$. The optimization algorithm made use of the link between network structure and spreading dynamics to find resections that led to a minimum seizure propagation by minimizing the efficiency of the seed, E_{seed}. The seed in this case was defined as the set of regions with (significantly) non-zero seed-probability, and the contribution of each ROI was weighted by its seed-probability (see methods section for more details). At the population level, $E_{\text{seed}}(R)$ decreased with the size of the resection for all patients (see an exemplary case in Supp. figure 4A-top, and the remaining cases in Supp. figure 5). To diminish differences due to seed extent and initial efficiency, we computed the normalized seed efficiency $e_R(\text{seed}) = E_0(\text{seed}) - E_R(\text{seed})$ (Supp. figure 4B-top for the population average). At the group level, the SF group showed a significantly larger $e_R(\text{seed})$ than the NSF group ($F(19) = 14.80$, $p = 10^{-32}$), although the effect of increasing the resection size was not significantly different ($F(19) = 0.74$, $p = 0.8$).

The actual effect of each resection on seizure propagation in the model was quantified using the SIR dynamics and 300 independent seed realizations. An exemplary case is shown in Supp. figure 4A-bottom, and the group average can be seen in Supp. figure 4B-middle. Seizure propagation depended heavily on the seed realization. A bi-stable regime emerged where, depending on the seed realization, the modelled seizures either propagated macroscopically or died locally. For each resection size we measured the normalized decrease in seizure propagation $\delta IR(R)$. At the group level, the SF group had a significantly smaller IR ($F(19) = 7.86$, $p = 10^{-16}$, Supp. figure 4B-middle), but there was no significant difference in the effect of increasing the resection size ($F(19) = 0.29$, $p \approx 1$).

We defined the normalized decrease in seizure propagation to compare between different patients as $\delta IR(R) = (IR_0 - IR_R)/IR_0$ (Supp. figure 4B-bottom). At a group level, the SF group presented significantly larger $\delta IR(R)$ for $S(R) > 1$ ($F(19) = 8.57$, $p = 2 \cdot 10^{-18}$), and again there was no significant difference in the effect of increasing the resection size between the groups ($F(19) = 0.27$, $p \approx 1$).

In order to compare the two groups systematically, we defined the optimal resection R_{op} as the one leading to a 90% decrease in seizure propagation, $\delta IR = 0.90$, and the disconnecting resection R_D as the smallest resection leading to seed disconnection, $e_{R_D} = 0$. We found that the SF group had smaller optimal resections (panel C-top) than the NSF group, but the difference was not significant (diff$= -5.73$, $rks = 81$ $p = 0.37$). The overlap between the optimal and actual resection was also larger, but not significantly so, for the SF group (panel D-top, diff$= 0.23$, $rks = 98$ $p = 0.20$). The disconnecting resection (panel E-top) was smaller, but not significantly so, for the SF than for the NSF group (diff$= -6.48$, $rks = 81$ $p = 0.37$). Either of these three variables could classify the two groups with good AUC results of 0.66, 0.71 and 0.66, respectively (mid subpanels of panels C, D and E). The confusion matrices corresponding to the optimal points (Youden criterion) are also shown in Supp. figure 4C,D,E. The classifications resulted in accuracy = 0.80, precision = 0.67, sensitivity = 0.50 and $F1 = 0.57$ for the size of optimal resections, accuracy = 0.67, precision = 0.44, sensitivity = 1.00 and $F1 = 0.62$ for the overlap of the optimal resections with the resection area, and accuracy = 0.80, precision = 0.67, sensitivity = 0.50 and $F1 = 0.57$ for the size of disconnecting resections.

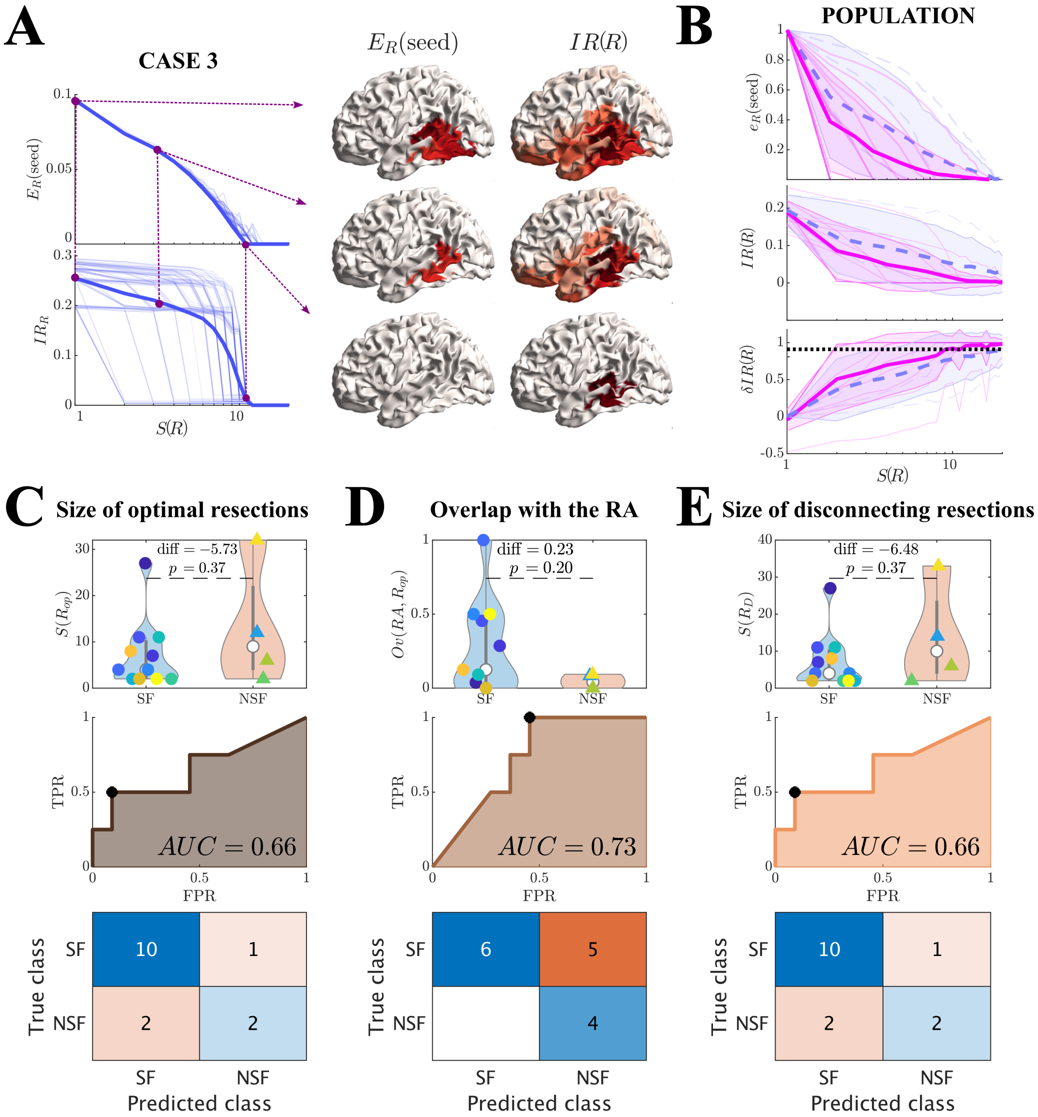

Supp. Figure 4: Optimization of virtual resections (modeling cohort). **A** Effect of resections of size $S(R)$ for an exemplary patient (case 3) as shown by the seed efficiency $E(\text{seed})_R$ (top) and total seizure propagation IR_R after the resection (bottom). Light lines in the top panel correspond to individual realizations of the optimization algorithm, and the solid wide line to the optimal resection of each size. This resection was used to quantify the effect on seizure propagation in the bottom panel. Light lines correspond each to a seed realization, whereas the solid wide line corresponds to the average over seed realizations. Brain plots on the right show $E(\text{seed})_R$ (left) and IR_R (right) for three exemplary resection sizes ($S(R) = 1, 3, S_D$), where S_D is the size of the disconnecting resection. **B** Group level analysis of the normalized seed efficiency $e(\text{seed})$, seizure propagation IR_R and normalized decrease in seizure propagation $\delta IR(R)$ after (optimal) resections of size $S(R)$. Blue dashed lines and pink solid lines stand respectively for NSF and SF patients. Wide lines indicate the group averages with uncertainties shown by the shaded areas, as given by the standard deviation. The dotted black line in the bottom panel indicates the 90% threshold used to define the optimal resection. **C, D, E** Group level comparison of the size of optimal resections (C), its overlap with the actual resection area (D) and the size of disconnecting resections (E). The top panels illustrate the violin plots for the SF and NSF groups, with significance results given by exact two-sided Wilcoxon ranksum tests. Data-points are coded with the same colour and marker type as in Supp. figure 1A. The middle panels show the corresponding ROC classification analyses, where FPR and TPR stand respectively for the false positive and true positive rates. The bottom panels show the confusion matrices corresponding to the optimal classification points (Youden criterion).

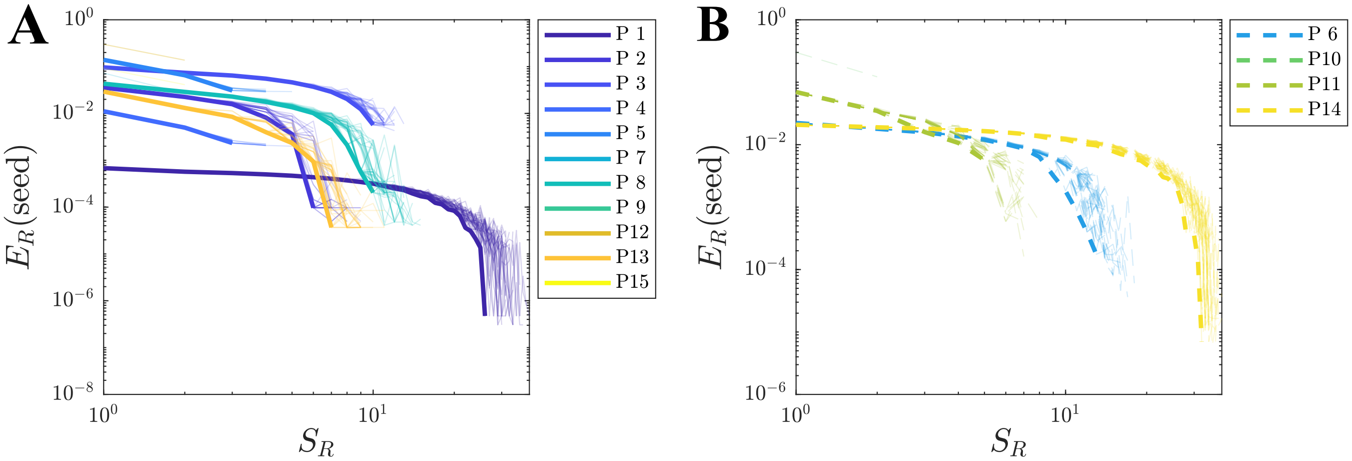

Supp. Figure 5: Optimization of virtual resection strategies for SF (panel A) and NSF (panel B) patients, showing the seed efficiency E_R (seed) after virtual resections of size $S(R)$. The light lines indicate individual realizations of the simulated annealing algorithm, whereas the thick lines indicate the best iteration (i.e. the one leading to the minimum E_R (seed). Each patient is shown by a different colour as indicated in the legends, with the same color code as Supp. figure 1A.

5.4 Disconnecting resection

The disconnecting resection R_D was defined as the smallest resection leading to seed disconnection, i.e. to $E_R(\text{seed}) = 0$. It is computationally much faster to obtain than R_{op} given that it does not require the simulation of the SIR spreading dynamics. As shown in Supp. figure 6E, the size of R_D is almost identical (correlation coefficient = 0.99) to the size of R_{op}.

We also show in Supp. figure 6 the correlation among the 4 model biomarkers (size of optimal and disconnecting resections $S(R_{op})$ and $S(R_D)$, overlap between the optimal and actual resection $Ov(RA, R_{op})$ and (model-based) effect of the actual resection $\delta IR(RA)$. Only $S(R_{op})$ and $S(R_D)$ were strongly correlated.

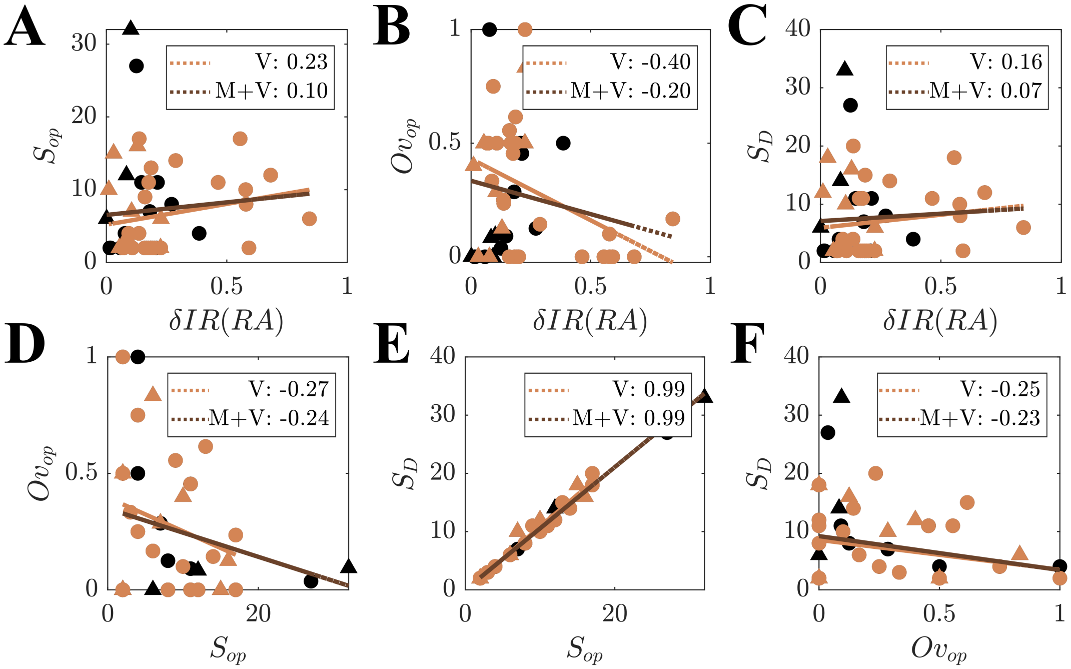

Supp. Figure 6: Pearson's correlation coefficient between each pair of model-derived variables: $\delta IR(RA)$, $S(R_{op})$, $Ov(RA, R_{op})$, $S(R_D)$. Black (dark) data-points stand for patients in the modeling cohorts, whereas orange (light) markers stand for patients in the validation cohort (thus the combined cohort includes all data-points). The orange (light) lines indicate the linear correlation for the validation cohort, and the brown (dark) ones stand for the combined cohort $V + M$. The correlation coefficients are indicated in the legends.

6 Modeling pipeline

The complete modeling pipeline is shown in Supp. figure 7. The study was performed in two steps. First, a retrospective study (modeling cohort) took place to fit the parameters of the different ESSES modules. For this cohort, the MEG and resection-area data had already been processed in a previous study [8]. The second step has been described in the main text (figure 2), and involves the validation cohort.

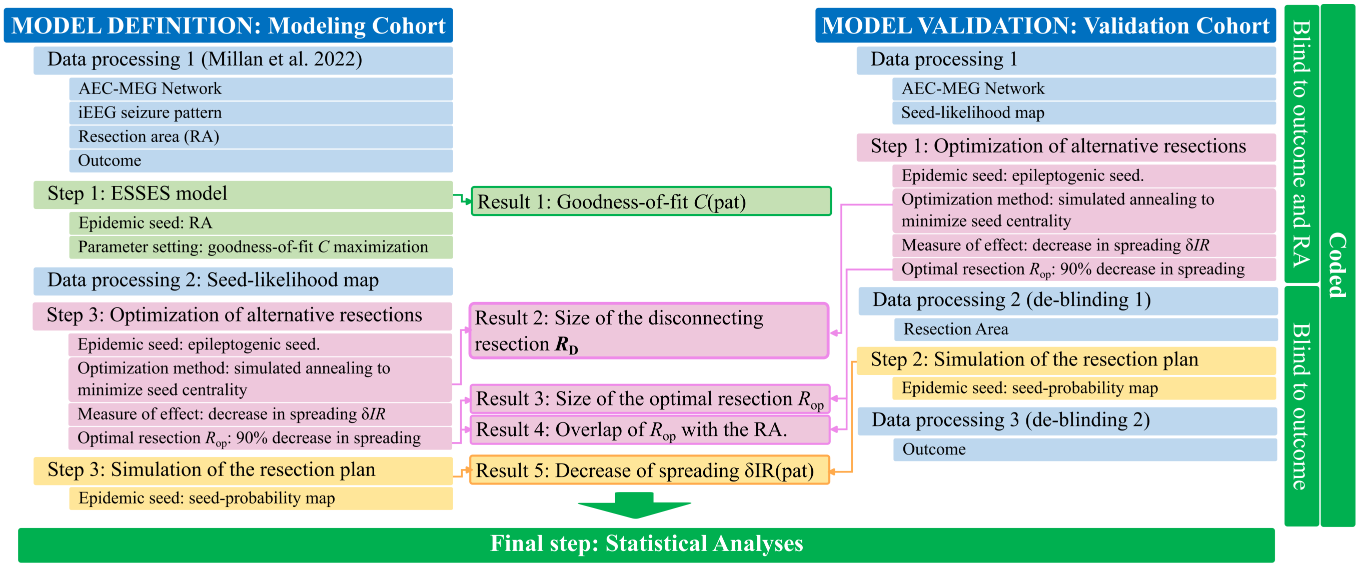

Supp. Figure 7: Methodological pipeline. The study was performed in two steps, with first a retrospective study to set-up the model parameters, followed by a pseudo-prospective application of the framework. The elements in this figure are color-coded according to the analysis step: data pre-processing (blue), model fit results (green, only modeling cohort), optimization of alternative resections (pink) and simulation of the resection plan (yellow). The final step consisted of the statistical analyses (group differences, patient classification and outcome prediction).

7 Simulation of the resection strategy: Modeling cohort

We made use of the population model and the seed-probability maps to simulate the effect of the clinical resection of each patient. The resection was simulated by disconnecting the resection area from the network, and the seed regions were derived probabilistically from the seed-probability maps. The effect of the resection was quantified as the relative decrease in seizure propagation due to the resection $\delta IR(RA) = (IR_0 - IR_R(RA))/IR_0$, where IR_0 and $IR_R(RA)$ stand respectively for the propagation before and after the resection (Supp. figure 8A). The seizure propagation and the effect of the resection depended strongly on the seed realization, so the same set of seeds were used before and after the resection to estimate $\delta IR(RA)$, and we considered 300 independent seed realizations to diminish noise effects.

The effect of the surgery was larger for the SF than for the NSF group (Supp. figure 8B), although the group difference was not significant ($\delta IR(RA, SF) - \delta IR(RA, NSF) = 0.14$, $rks = 98$, $p = 0.22$). A ROC analysis using $\delta IR(RA)$ to classify the two groups found an AUC$= 0.73$ (Supp. figure 8C). The confusion matrix corresponding to the optimal classification point (Youden criterion, Supp. figure 8D) shows an accuracy of 0.73, precision $= 0.50$, sensitivity $= 1.00$ and $F1 = 0.67$. Again, in this case the classification analysis was able to identify all NSF patients correctly.

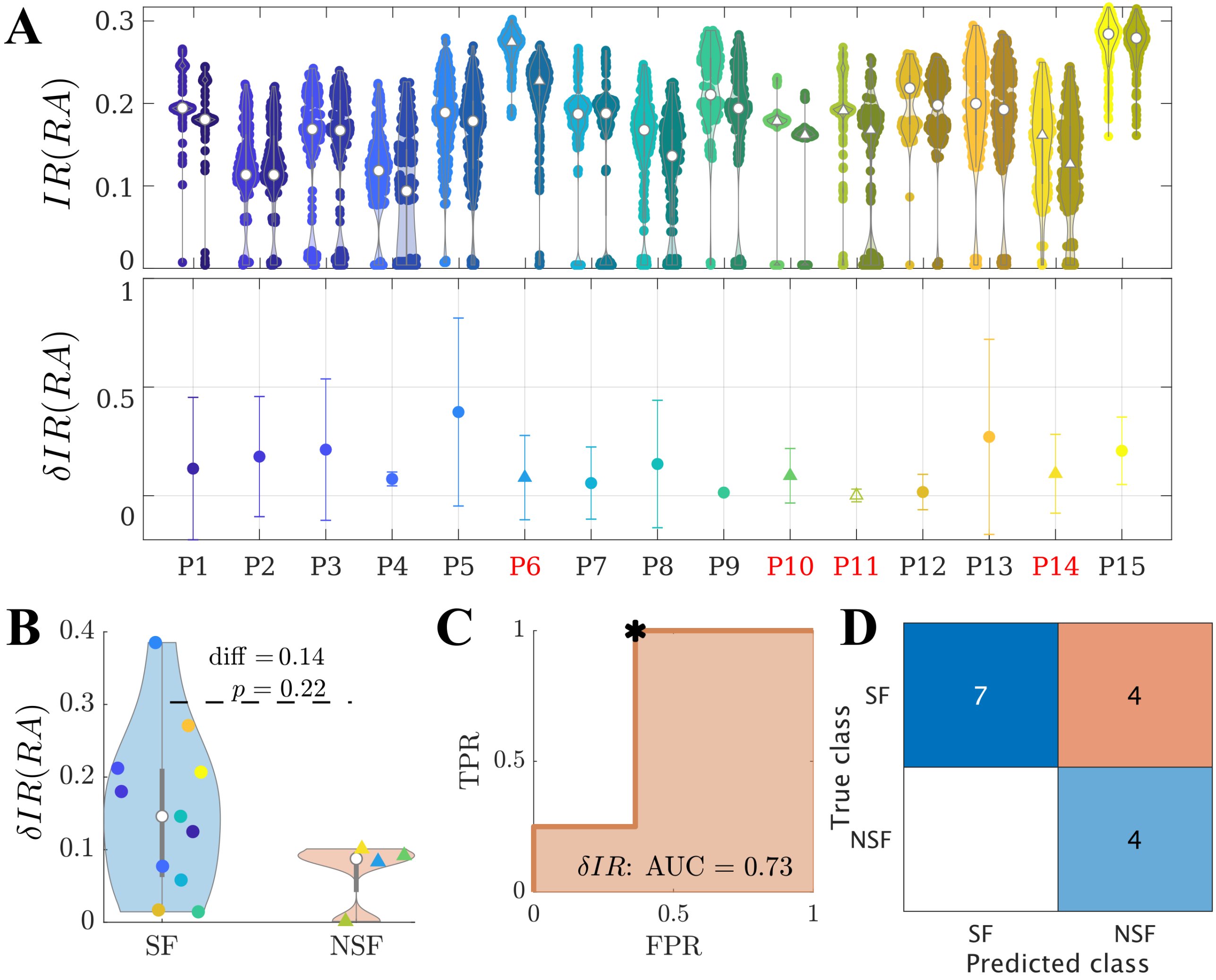

Supp. Figure 8: Simulation of the resective surgery (modelling cohort). **A** Effect of the virtual resections of the resection area in the model. The top panel shows the total seizure propagation IR before and after the resection for each patient (respectively left and right violin plots for each x-tick), for 300 independent realizations of the seed regions. The white markers (respectively circles for SF and triangles for NSF cases) show the average over the seed realizations. NSF cases are also highlighted by red labels on the x-axis. The bottom panel shows the relative decrease in seizure propagation $\delta IR(RA)$ for each patient, as given by the average (data-points) and standard deviation (error bars). The colorcode is as in Supp. figure 1A. **B, C, D** Virtual resections of the resection area had a (not significantly) larger effect for the SF than NSF group, as shown by the violin plots of $\delta IR(RA)$ in panel B. A ROC classification analysis of the two groups based on $\delta IR(RA)$ showed an AUC$= 0.73$ (panel C). TPR and FPR respectively stand for the true positive (NSF patients classified as NSF) and false positive (SF patients classified as NSF) rates. The confusion matrix corresponding to the optimal operating point (Youden criterion) is shown in panel D. For this threshold we found accuracy $= 0.73$, precision $= 0.50$, sensitivity $= 1.00$, and $F1 = 0.67$.

8 Classification results: Modeling cohort.

The results of the classification results for the modeling cohort are shown in Supp. table 4.

Variable	True negatives: SF	True positives: NSF	Acc.	Prec.	Sensitivity	$F1$	AUC
$\bar{C}$	8/11 (= 0.73)	4/4 (= 1.00)	0.80	0.57	1.00	0.71	0.79
$S(R_{op})$	10/11 (= 0.91)	2/4 (= 0.50)	0.80	0.67	0.50	0.57	0.66
$Ov(RA, R_{op})$	6/11 (= 0.55)	4/4 (= 1.00)	0.67	0.44	1.00	0.62	0.73
$\delta IR(RA)$	7/11 (= 0.64)	4/4 (= 1.00)	0.73	0.50	1.00	0.67	0.73

Supp. Table 4: Results of the classification analyses for the modeling cohort. Results correspond to the optimal points of the ROC curves according to the Youden criterion to account for class imbalance. For each group (SF, NSF), we show the number of correctly identified cases by absolute number and relative frequency. The remaining columns correspond respectively to the accuracy (Acc.), precision (Prec.), sensitivity, F1 statistic and area under the curve (AUC).

References

[1] S. Taulu and J. Simola. "Spatiotemporal signal space separation method for rejecting nearby interference in MEG measurements". *Physics in Medicine & Biology* 51.7 (2006), p. 1759.

[2] S. Taulu and R. Hari. "Removal of magnetoencephalographic artifacts with temporal signal-space separation: demonstration with single-trial auditory-evoked responses". *Human Brain Mapping* 30.5 (2009), pp. 1524–1534.

[3] A. Hillebrand et al. "Feasibility of clinical magnetoencephalography (MEG) functional mapping in the presence of dental artefacts". *Clinical Neurophysiology* 124.1 (2013), pp. 107–113.

[4] A. Hillebrand et al. "Frequency-dependent functional connectivity within resting-state networks: an atlas-based MEG beamformer solution". *Neuroimage* 59.4 (2012), pp. 3909–3921.

[5] A. Hillebrand et al. "Direction of information flow in large-scale resting-state networks is frequency-dependent". *Proceedings of the National Academy of Sciences* 113.14 (2016), pp. 3867–3872.

[6] L. Fan et al. "The human brainnetome atlas: a new brain atlas based on connectional architecture". *Cerebral Cortex* 26.8 (2016), pp. 3508–3526.

[7] *Brainwave.* https://home.kpn.nl/stam7883/brainwave.html.

[8] A. P. Millán et al. "The role of epidemic spreading in seizure dynamics and epilepsy surgery". *Network Neuroscience* 7.2 (2023), pp. 811–843.

[9] R. Pastor-Satorras et al. "Epidemic processes in complex networks". *Reviews of Modern Physics* 87.3 (2015), pp. 925–979.

[10] B. Prasse and P. Van Mieghem. "The viral state dynamics of the discrete-time NIMFA epidemic model". *IEEE Transactions on Network Science and Engineering* 7.3 (2019), pp. 1667–1674.

[11] A. P. Millán et al. "Epidemic models characterize seizure propagation and the effects of epilepsy surgery in individualized brain networks based on MEG and invasive EEG recordings". *Scientific Reports* 12.1 (2022), pp. 1–20.

Lobe	Sublobes
Frontal	Orbital<br>Basal<br>Parasagital<br>Periventricular<br>Lateral<br>Operculum
Temporal	Hippocampus<br>Amygdala<br>Uncus<br>Anterior-neocortical<br>Posterior-neocortical<br>Gyrus-parahippocampalis
Insula	Anterior<br>Posterior
Central	Central
Parietal	Parietal
Occipital	Occipital

Lobe	Gyrus	Anatomical and modified Cyto-architectonic descriptions	Label ID.L	Label ID.R	Castor sublobe
**Frontal Lobe**	SFG, Superior Frontal Gyrus	*A8m, medial area 8*	1	2	frontal parasagittal
		A8dl, dorsolateral area 8	3	4	frontal lateral
		A9l, lateral area 9	5	6	frontal lateral
		A6dl, dorsolateral area 6	7	8	frontal lateral
		A6m, medial area 6	9	10	frontal parasagittal
		A9m,medial area 9	11	12	frontal parasagittal
		A10m, medial area 10	13	14	frontal parasagittal
	MFG, Middle Frontal Gyrus	*A9/46d, dorsal area 9/46*	15	16	frontal lateral
		IFJ, inferior frontal junction	17	18	frontal lateral
		A46, area 46	19	20	frontal lateral
		A9/46v, ventral area 9/46	21	22	frontal lateral
		A8vl, ventrolateral area 8	23	24	frontal lateral
		A6vl, ventrolateral area 6	25	26	frontal lateral
		A10l, lateral area10	27	28	frontal lateral
	IFG, Inferior Frontal Gyrus	*A44d,dorsal area 44*	29	30	frontal lateral
		IFS, inferior frontal sulcus	31	32	frontal lateral
		A45c, caudal area 45	33	34	frontal lateral
		A45r, rostral area 45	35	36	frontal lateral
		A44op, opercular area 44	37	38	frontal operculum
		A44v, ventral area 44	39	40	frontal lateral
	OrG, Orbital Gyrus	*A14m, medial area 14*	41	42	frontal orbital
		A12/47o, orbital area 12/47	43	44	frontal basal
		A11l, lateral area 11	45	46	frontal orbital
		A11m, medial area 11	47	48	frontal orbital
		A13, area 13	49	50	frontal orbital
		A12/47l, lateral area 12/47	51	52	frontal basal
	PrG, Precentral Gyrus	*A4hf, area 4(head and face region)*	53	54	central
		A6cdl, caudal dorsolateral area 6	55	56	central
		A4ul, area 4(upper limb region)	57	58	central
		A4t, area 4(trunk region)	59	60	central
		A4tl, area 4(tongue and larynx region)	61	62	central
		A6cvl, caudal ventrolateral area 6	63	64	frontal lateral

	PCL, Paracentral Lobule	*A1/2/3ll, area1/2/3 (lower limb region)*	65	66	central
		A4ll, area 4, (lower limb region)	67	68	central
**Temporal Lobe**	STG, Superior Temporal Gyrus	*A38m, medial area 38*	69	70	temporal anterior neocortical
		A41/42, area 41/42	71	72	temporal posterior neocortical
		TE1.0 and TE1.2	73	74	temporal anterior neocortical
		A22c, caudal area 22	75	76	temporal posterior neocortical
		A38l, lateral area 38	77	78	temporal anterior neocortical
		A22r, rostral area 22	79	80	temporal anterior neocortical
	MTG, Middle Temporal Gyrus	*A21c, caudal area 21*	81	82	temporal posterior neocortical
		A21r, rostral area 21	83	84	temporal anterior neocortical
		A37dl, dorsolateral area37	85	86	temporal posterior neocortical
		aSTS, anterior superior temporal sulcus	87	88	temporal anterior neocortical
	ITG, Inferior Temporal Gyrus	*A20iv, intermediate ventral area 20*	89	90	temporal anterior neocortical
		A37elv, extreme lateroventral area37	91	92	temporal posterior neocortical
		A20r, rostral area 20	93	94	temporal anterior neocortical
		A20il, intermediate lateral area 20	95	96	temporal anterior neocortical
		A37vl, ventrolateral area 37	97	98	temporal posterior neocortical
		A20cl, caudolateral of area 20	99	100	temporal posterior neocortical
		A20cv, caudoventral of area 20	101	102	temporal anterior neocortical
	FuG, Fusiform Gyrus	*A20rv, rostroventral area 20*	103	104	temporal neocortical
		A37mv, medioventral area37	105	106	temporal posterior neocortical
		A37lv, lateroventral area37	107	108	temporal posterior neocortical
	PhG, Parahippocampal Gyrus	*A35/36r, rostral area 35/36*	109	110	temporal parahippocampal
		A35/36c, caudal area 35/36	111	112	temporal parahippocampal
		TL, area TL (lateral PPHC, posterior parahippocampal gyrus)	113	114	temporal parahippocampal
		A28/34, area 28/34 (EC, entorhinal cortex)	115	116	temporal parahippocampal
		TI, area TI(temporal agranular insular cortex)	117	118	temporal parahippocampal
		TH, area TH (medial PPHC)	119	120	temporal parahippocampal
	pSTS, posterior Superior Temporal Sulcus	*rpSTS, rostroposterior superior temporal sulcus*	121	122	temporal posterior neocortical
		cpSTS, caudoposterior superior temporal sulcus	123	124	temporal posterior neocortical
	SPL, Superior Parietal Lobule	*A7r, rostral area 7*	125	126	parietal
		A7c, caudal area 7	127	128	parietal
		A5l, lateral area 5	129	130	parietal
		A7pc, postcentral area 7	131	132	central

**Parietal Lobe**		*A7ip, intraparietal area 7(hIP3)*	133	134	parietal
	IPL, Inferior Parietal Lobule	*A39c, caudal area 39(PGp)*	135	136	parietal
		A39rd, rostrodorsal area 39(Hip3)	137	138	parietal
		A40rd, rostrodorsal area 40(PFt)	139	140	parietal
		A40c, caudal area 40(PFm)	141	142	parietal
		A39rv, rostroventral area 39(PGa)	143	144	parietal
		A40rv, rostroventral area 40(PFop)	145	146	parietal
	Pcun, Precuneus	*A7m, medial area 7(PEp)*	147	148	parietal
		A5m, medial area 5(PEm)	149	150	parietal
		dmPOS, dorsomedial parietooccipital sulcus(PEr)	151	152	parietal
		A31, area 31 (Lc1)	153	154	parietal
	PoG, Postcentral Gyrus	*A1/2/3ulhf, area 1/2/3(upper limb, head and face region)*	155	156	central
		A1/2/3tonIa, area 1/2/3(tongue and larynx region)	157	158	central
		A2, area 2	159	160	parietal
		A1/2/3tru, area1/2/3(trunk region)	161	162	central
**Insular Lobe**	INS, Insular Gyrus	*G, hypergranular insula*	163	164	posterior insula
		vIa, ventral agranular insula	165	166	anterior insula
		dIa, dorsal agranular insula	167	168	anterior insula
		vId/vIg, ventral dysgranular and granular insula	169	170	anterior insula
		dIg, dorsal granular insula	171	172	anterior insula
		dId, dorsal dysgranular insula	173	174	anterior insula
**Limbic Lobe**	CG, Cingulate Gyrus	*A23d, dorsal area 23*	175	176	parietal
		A24rv, rostroventral area 24	177	178	frontal parasagittal
		A32p, pregenual area 32	179	180	frontal parasagittal
		A23v, ventral area 23	181	182	occipital
		A24cd, caudodorsal area 24	183	184	frontal parasagittal
		A23c, caudal area 23	185	186	central
		A32sg, subgenual area 32	187	188	frontal parasagittal
**Occipital Lobe**	MVOcC, MedioVentral Occipital Cortex	*cLinG, caudal lingual gyrus*	189	190	occipital
		rCunG, rostral cuneus gyrus	191	192	occipital
		cCunG, caudal cuneus gyrus	193	194	occipital
		rLinG, rostral lingual gyrus	195	196	occipital
		vmPOS,ventromedial parietooccipital sulcus	197	198	occipital
		mOccG, middle occipital gyrus	199	200	occipital

Lobe	LOcC, lateral Occipital Cortex	*V5/MT+, area V5/MT+*	201	202	occipital
		OPC, occipital polar cortex	203	204	occipital
		iOccG, inferior occipital gyrus	205	206	occipital
		msOccG, medial superior occipital gyrus	207	208	occipital
		lsOccG, lateral superior occipital gyrus	209	210	occipital
**Subcortical Nuclei**	Amyg, Amygdala	*mAmyg, medial amygdala*	211	212	amygdala
		lAmyg, lateral amygdala	213	214	amygdala
	Hipp, Hippocampus	*rHipp, rostral hippocampus*	215	216	hippocampus
		cHipp, caudal hippocampus	217	218	hippocampus
	BG, Basal Ganglia	*vCa, ventral caudate*	219	220	frontal periventricular
		GP, globus pallidus	221	222	frontal periventricular
		NAC, nucleus accumbens	223	224	frontal periventricular
		vmPu, ventromedial putamen	225	226	frontal periventricular
		dCa, dorsal caudate	227	228	frontal periventricular
		dlPu, dorsolateral putamen	229	230	frontal periventricular
	Tha, Thalamus	*mPFtha, medial pre-frontal thalamus*	231	232	not defined
		mPMtha, pre-motor thalamus	233	234	not defined
		Stha, sensory thalamus	235	236	not defined
		rTtha, rostral temporal thalamus	237	238	not defined
		PPtha, posterior parietal thalamus	239	240	not defined
		Otha, occipital thalamus	241	242	not defined
		cTtha, caudal temporal thalamus	243	244	not defined
		lPFtha, lateral pre-frontal thalamus	245	246	not defined

* Columns A-E are provided by the Brainnetome atlas library at https://atlas.brainnetome.org/bnatlas.html
